# Supplementary material for: Population Structure and Genetic Diversity of Italian Beef Breeds as a Tool for Planning Conservation and Selection Strategies
Source: Animals (Basel). 2019 Oct 29;9(11):880. doi: 10.3390/ani9110880 (PMC6912484; doi:10.3390/ani9110880)
Supplement: Supplementary file 1 [file animals-09-00880-s001.zip › Supp_Fig1.pdf]

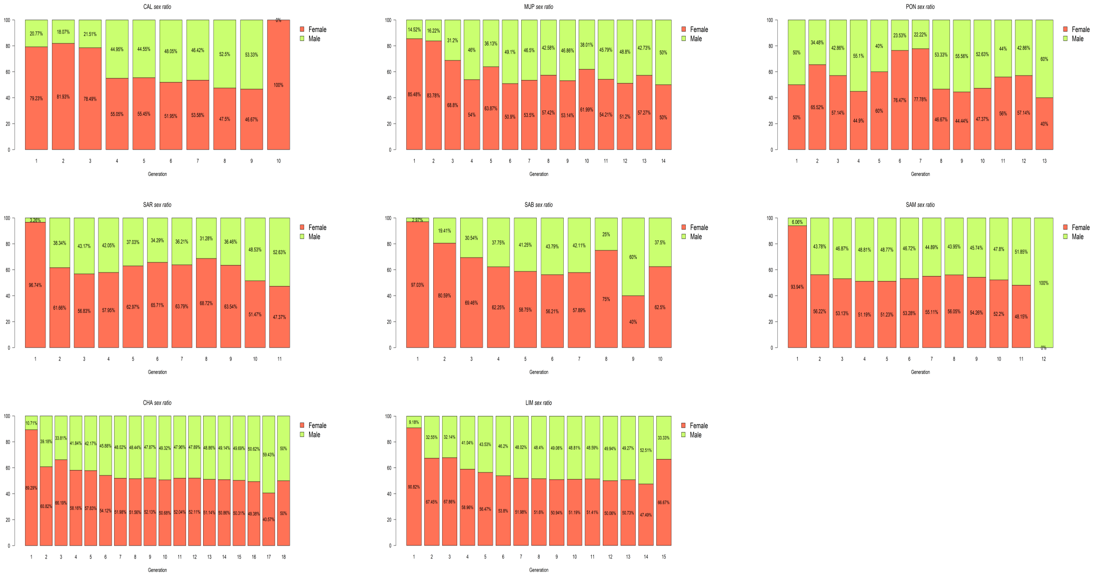
CAL sex ratio
Female
Male
20.77% 18.07% 21.51% 44.95% 44.55% 48.05% 46.42% 52.5% 53.33% 0%
79.23% 81.93% 78.49% 55.05% 55.45% 51.95% 53.58% 47.5% 46.67% 100%
Generation
MUP sex ratio
14.52% 16.22% 31.2% 46% 36.13% 49.1% 46.5% 42.58% 46.86% 38.01% 45.79% 48.8% 42.73% 50%
85.48% 83.78% 68.8% 54% 63.87% 50.9% 53.5% 57.42% 53.14% 61.99% 54.21% 51.2% 57.27% 50%
PON sex ratio
50% 34.48% 42.86% 55.1% 40% 23.53% 22.22% 53.33% 55.56% 52.63% 44% 42.86% 60%
50% 65.52% 57.14% 44.9% 60% 76.47% 77.78% 46.67% 44.44% 47.37% 56% 57.14% 40%
SAR sex ratio
3.26% 38.34% 43.17% 42.05% 37.03% 34.29% 36.21% 31.28% 36.46% 48.53% 52.63%
96.74% 61.66% 56.83% 57.95% 62.97% 65.71% 63.79% 68.72% 63.54% 51.47% 47.37%
SAB sex ratio
2.97% 19.41% 30.54% 37.75% 41.25% 43.79% 42.11% 25% 60% 37.5%
97.03% 80.59% 69.46% 62.25% 58.75% 56.21% 57.89% 75% 40% 62.5%
SAM sex ratio
6.06% 43.78% 46.87% 48.81% 48.77% 46.72% 44.89% 43.95% 45.74% 47.8% 51.85% 100%
93.94% 56.22% 53.13% 51.19% 51.23% 53.28% 55.11% 56.05% 54.26% 52.2% 48.15% 0%
CHA sex ratio
10.71% 39.18% 33.81% 41.84% 42.17% 45.88% 48.02% 48.44% 47.87% 49.32% 47.96% 47.89% 48.86% 49.14% 49.69% 50.62% 59.43% 50%
89.29% 60.82% 66.19% 58.16% 57.83% 54.12% 51.98% 51.56% 52.13% 50.68% 52.04% 52.11% 51.14% 50.86% 50.31% 49.38% 40.57% 50%
LIM sex ratio
9.18% 32.55% 32.14% 41.04% 43.53% 46.2% 48.02% 48.4% 49.06% 48.81% 48.59% 49.94% 49.27% 52.51% 33.33%
90.82% 67.45% 67.86% 58.96% 56.47% 53.8% 51.98% 51.6% 50.94% 51.19% 51.41% 50.06% 50.73% 47.49% 66.67%
