# Supplementary figures and images for: Population Structure and Genetic Diversity of Italian Beef Breeds as a Tool for Planning Conservation and Selection Strategies
Source: Animals (Basel). 2019 Oct 29;9(11):880. doi: 10.3390/ani9110880 (PMC6912484; doi:10.3390/ani9110880)

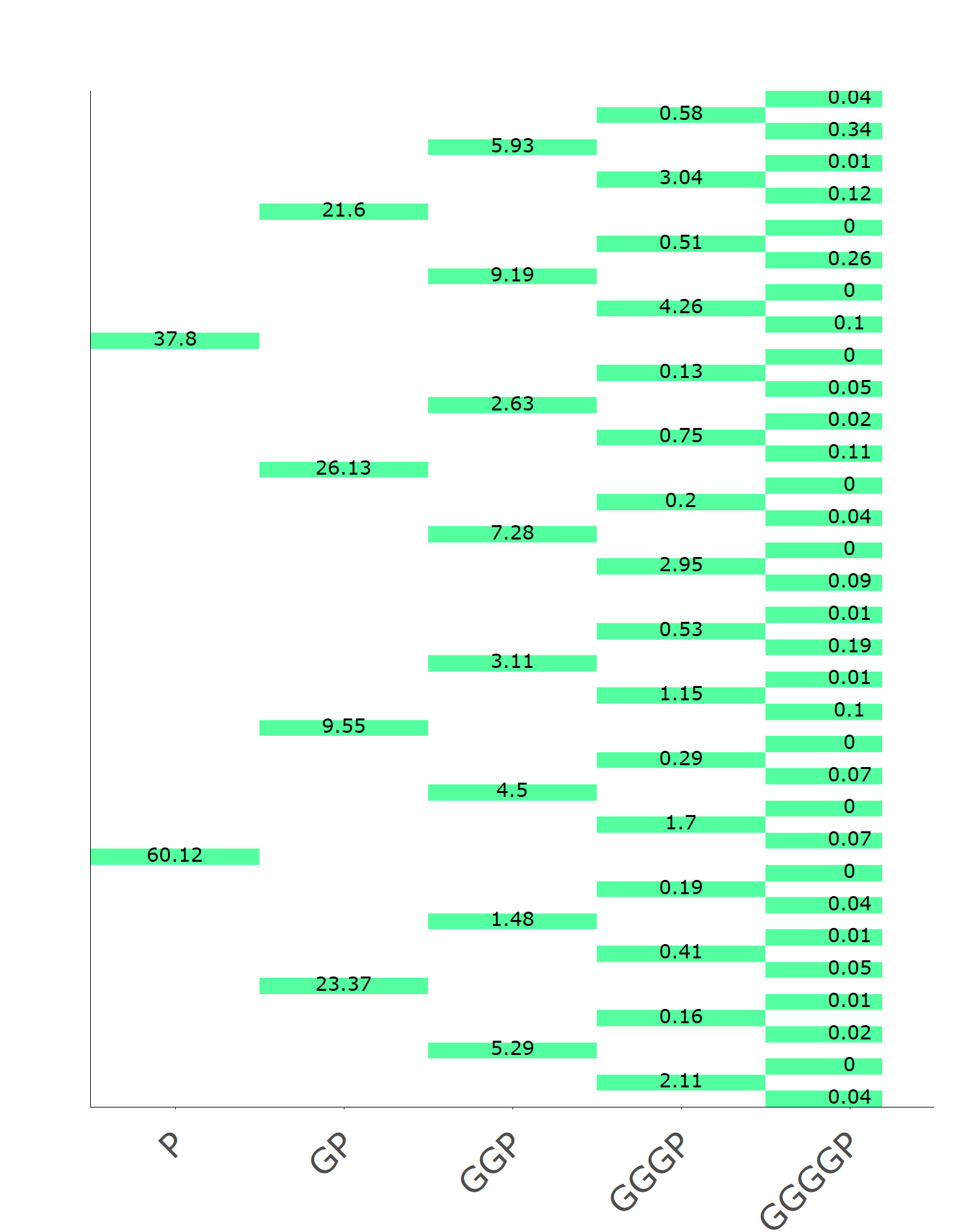

Supplement: Supplementary file 1 [file animals-09-00880-s001.zip › Suppl_F5_SAB.png]

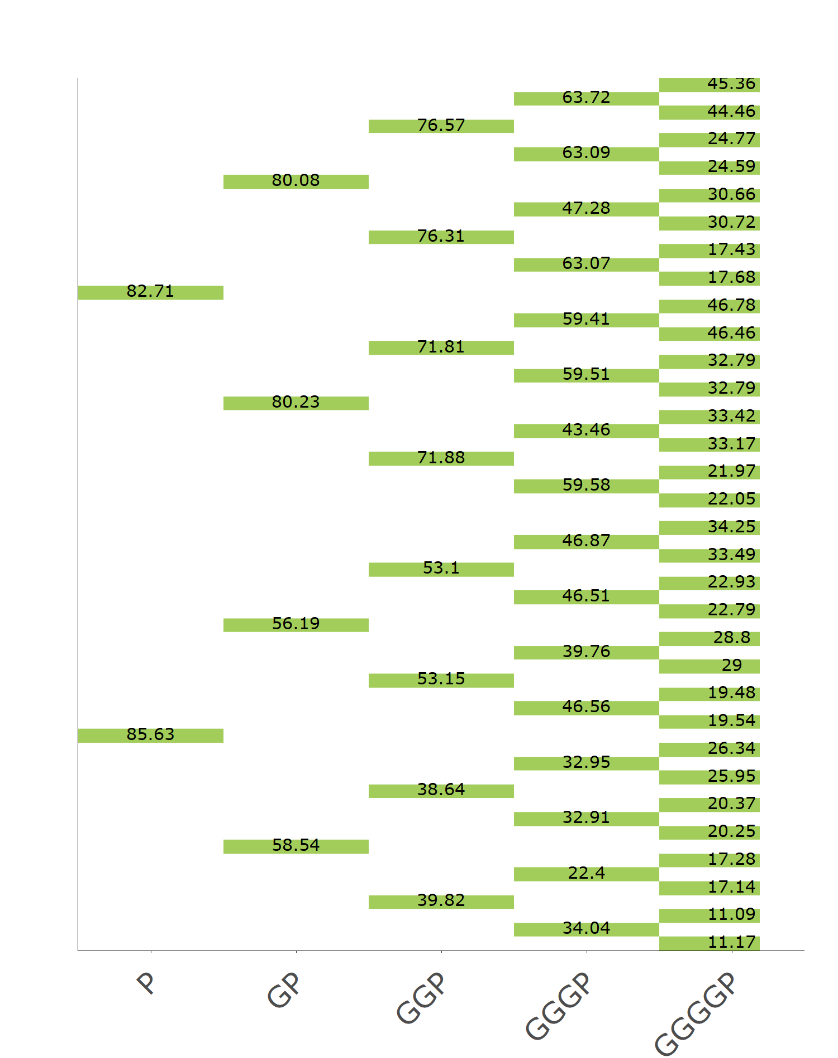

Supplement: Supplementary file 1 [file animals-09-00880-s001.zip › Suppl_F5_LIM.png]

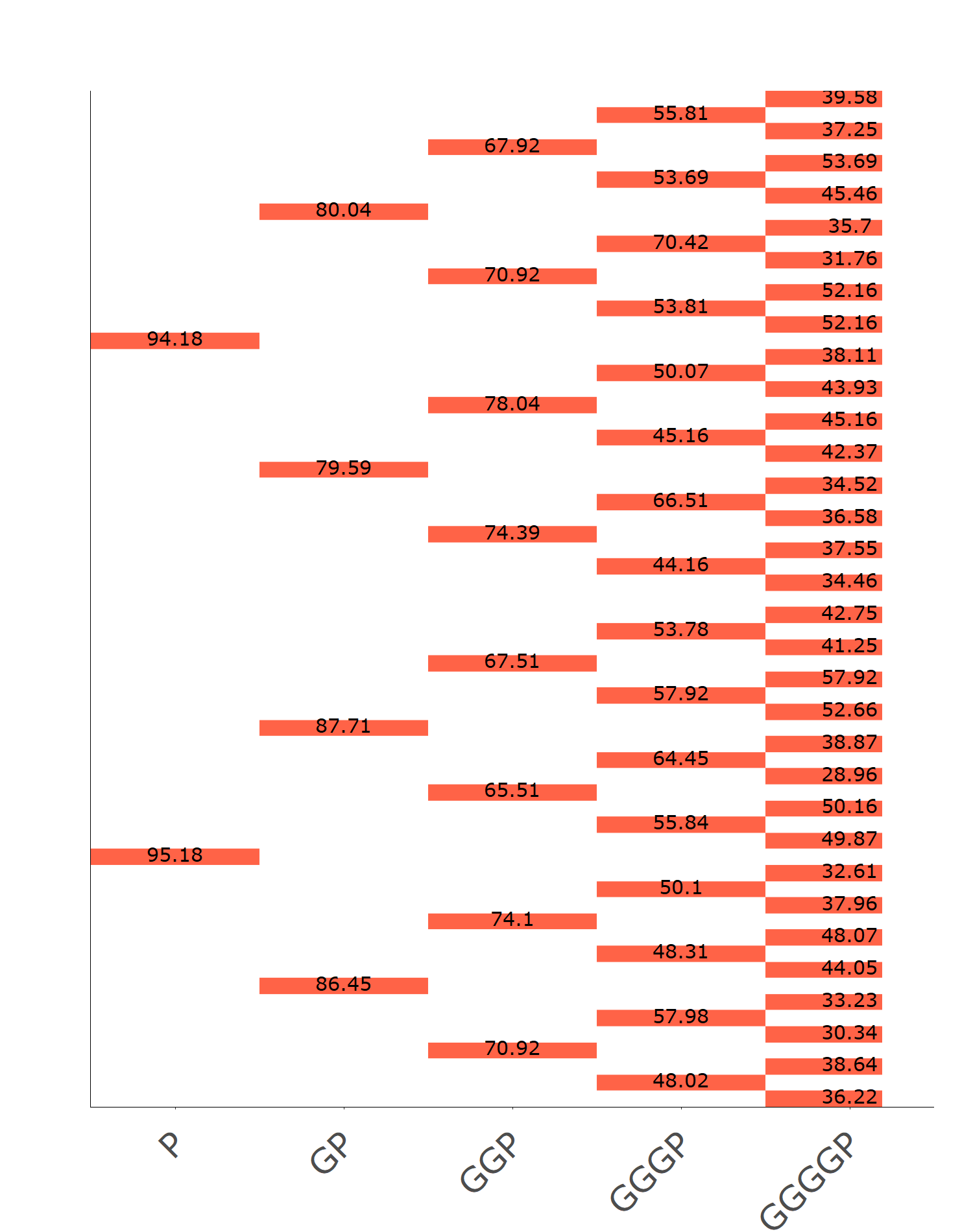

Supplement: Supplementary file 1 [file animals-09-00880-s001.zip › Suppl_F5_MUP.png]

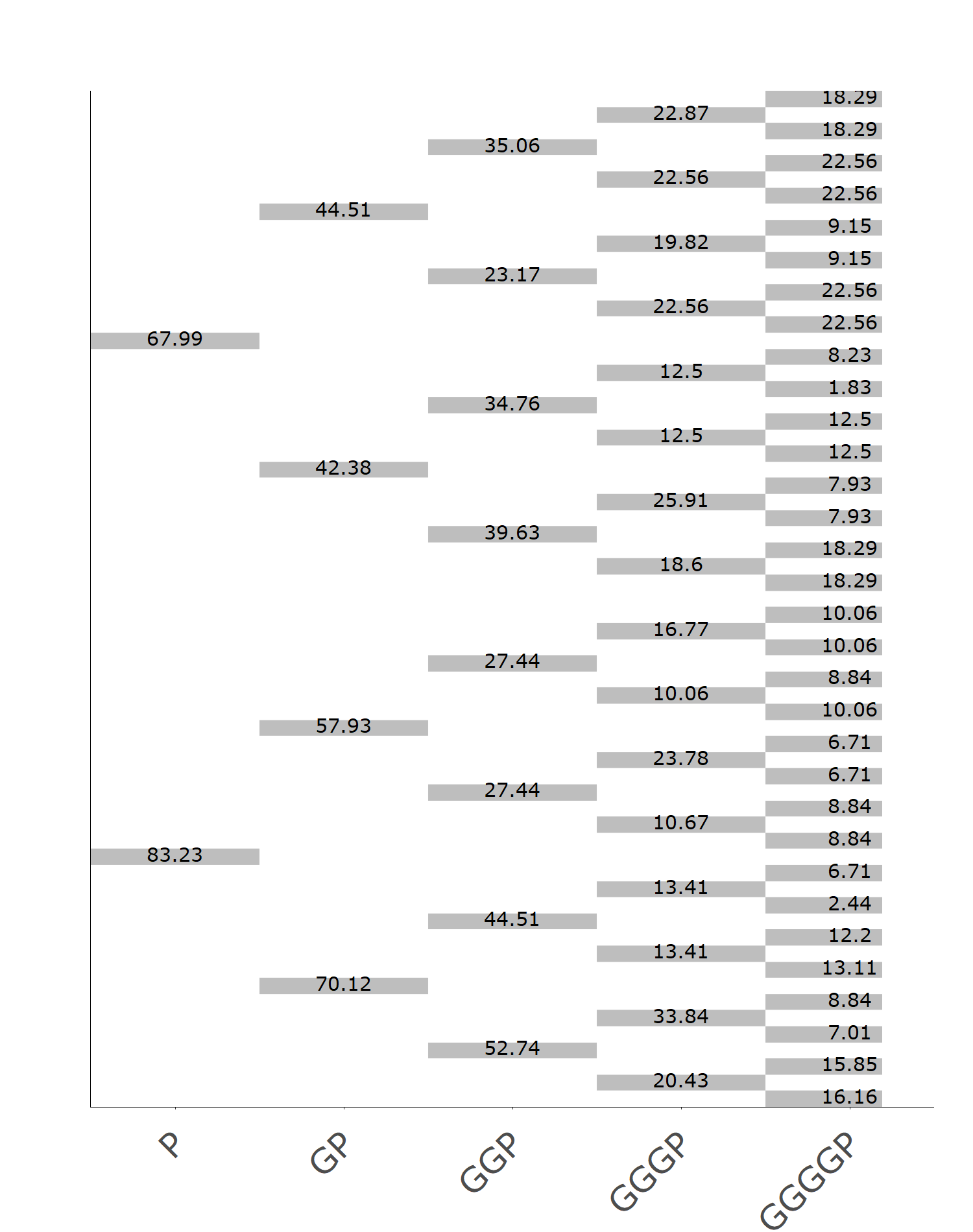

Supplement: Supplementary file 1 [file animals-09-00880-s001.zip › Suppl_F5_PON.png]

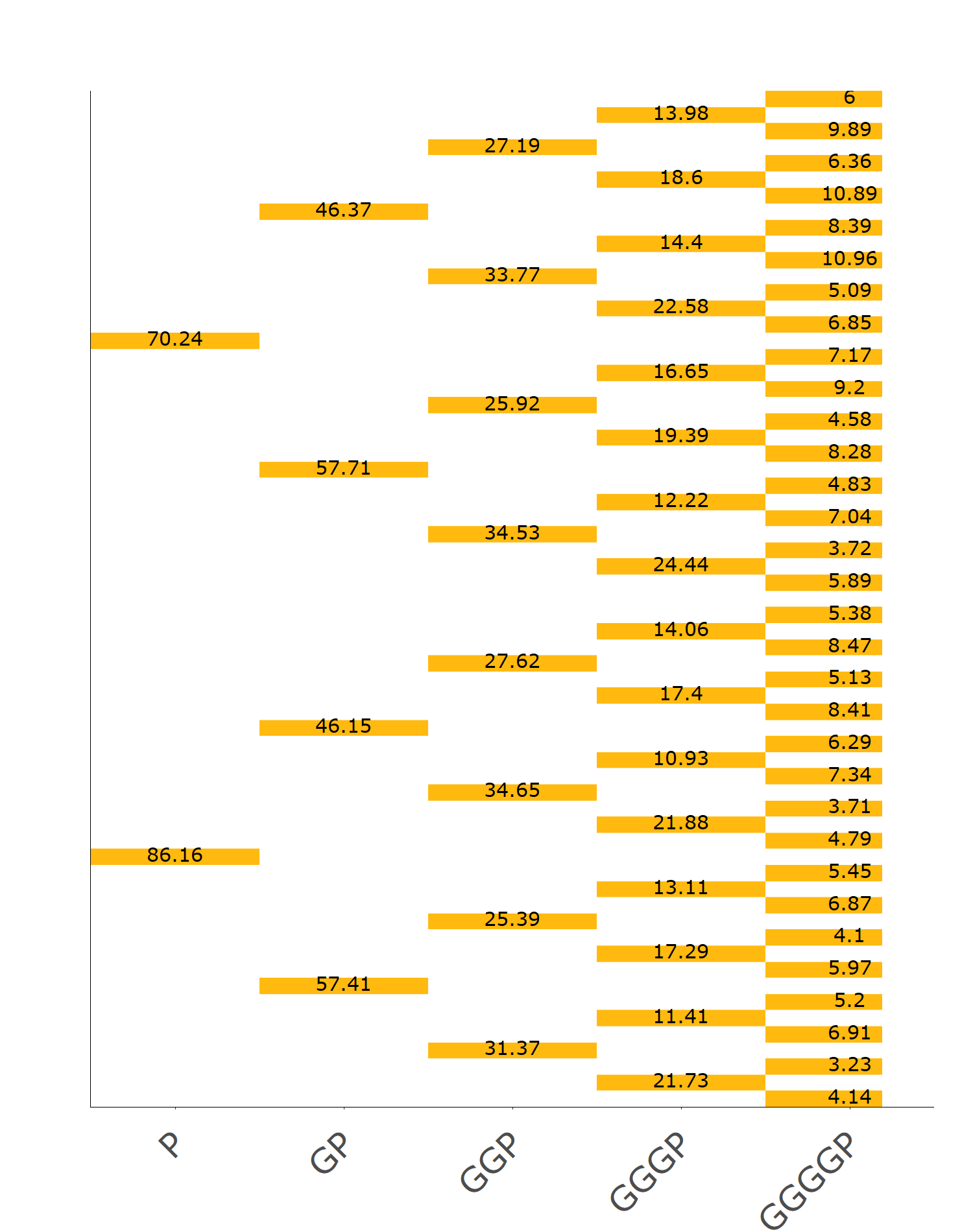

Supplement: Supplementary file 1 [file animals-09-00880-s001.zip › Suppl_F5_SAM.png]

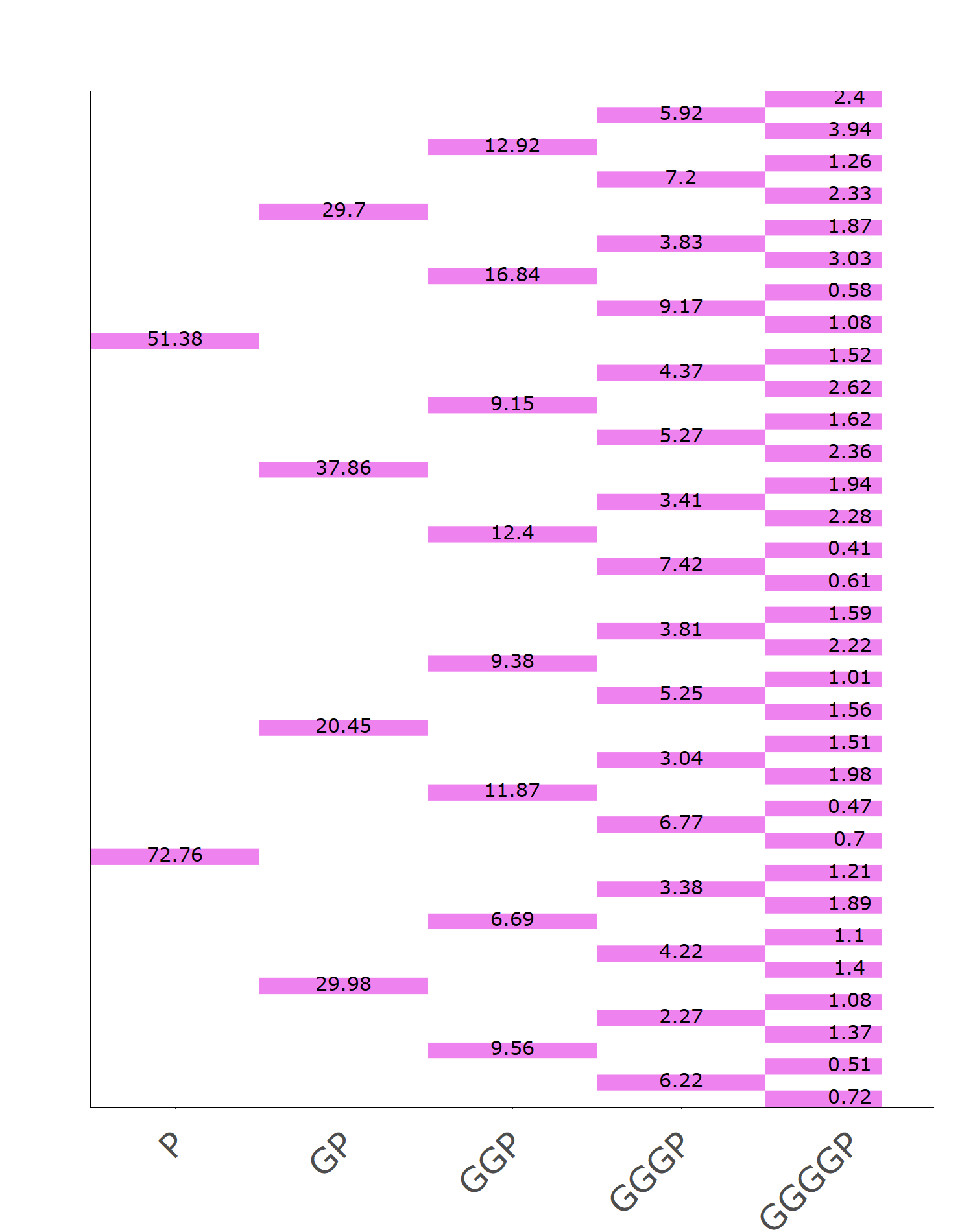

Supplement: Supplementary file 1 [file animals-09-00880-s001.zip › Suppl_F5_SAR.png]

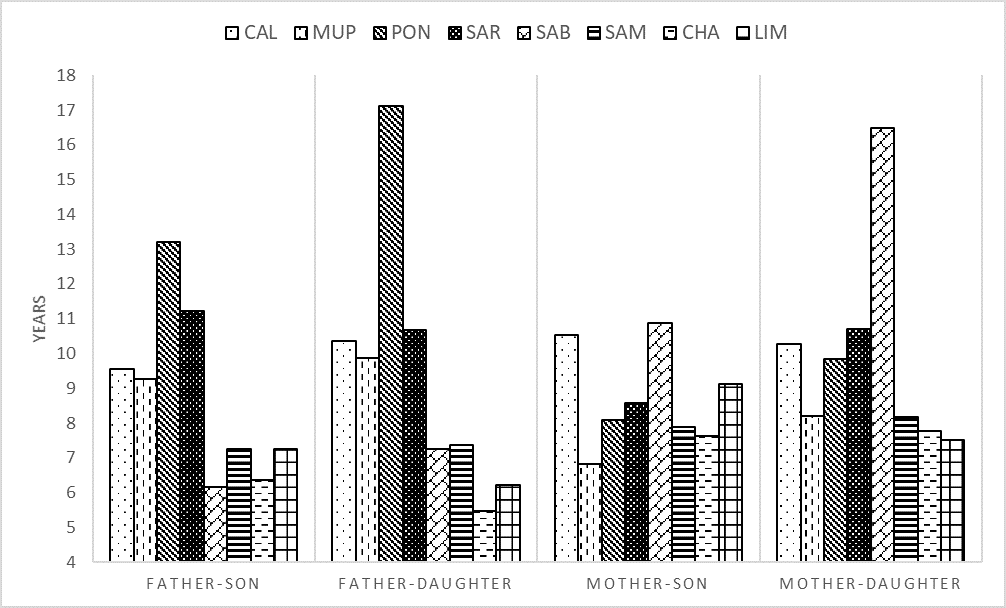

Supplement: Supplementary file 1 [file animals-09-00880-s001.zip › Supp_Fig4.png]

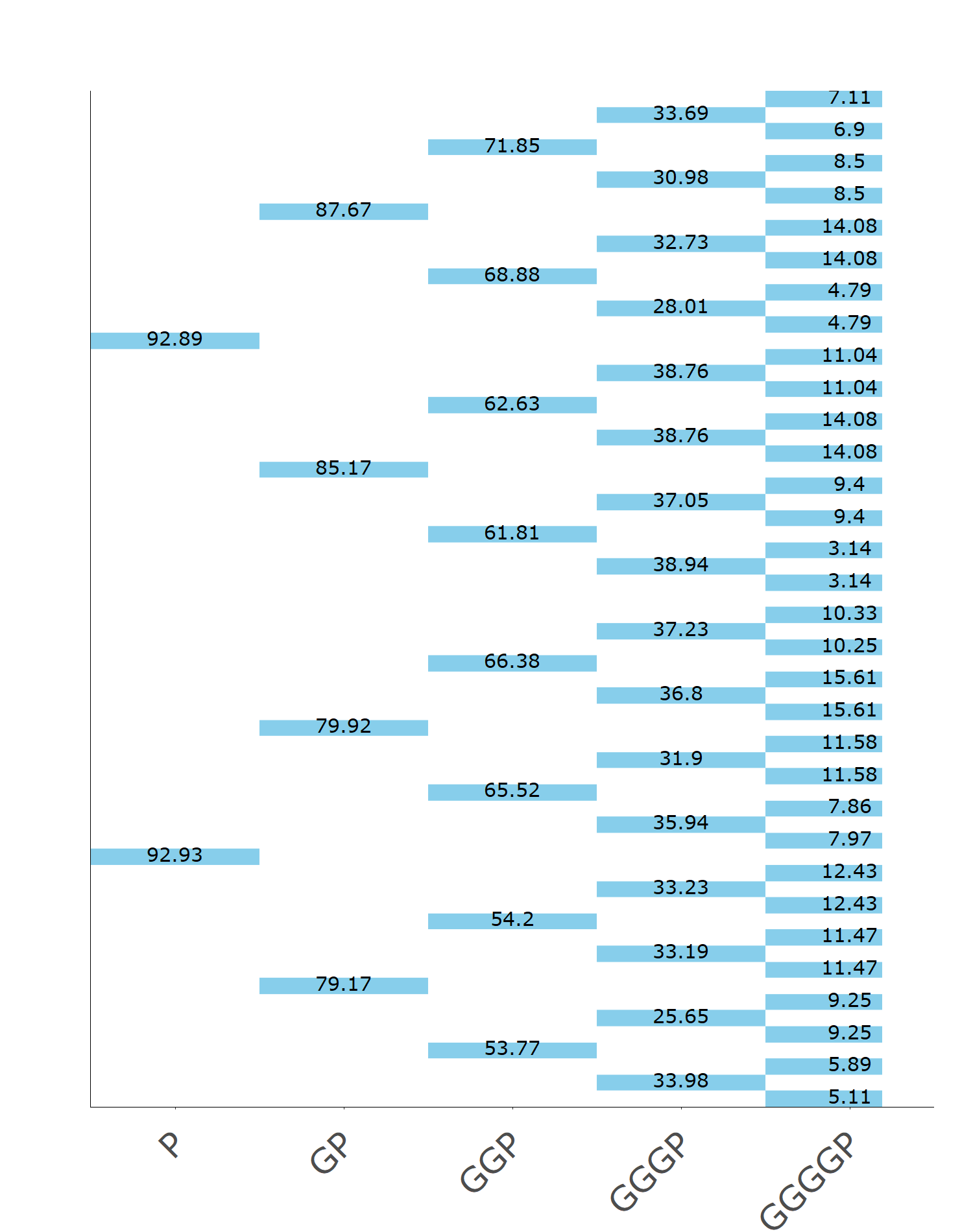

Supplement: Supplementary file 1 [file animals-09-00880-s001.zip › Suppl_f5_CAL.png]

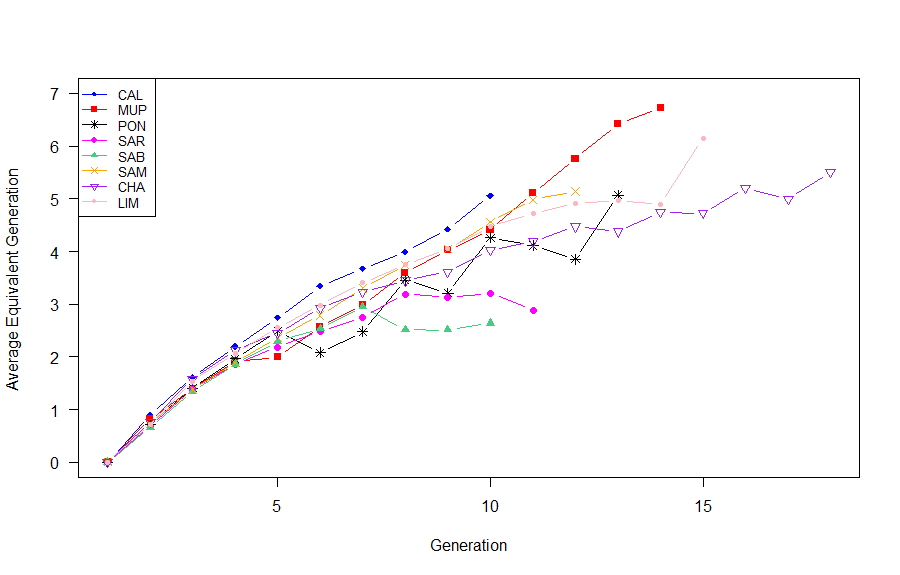

Supplement: Supplementary file 1 [file animals-09-00880-s001.zip › Supp_Fig3.png]

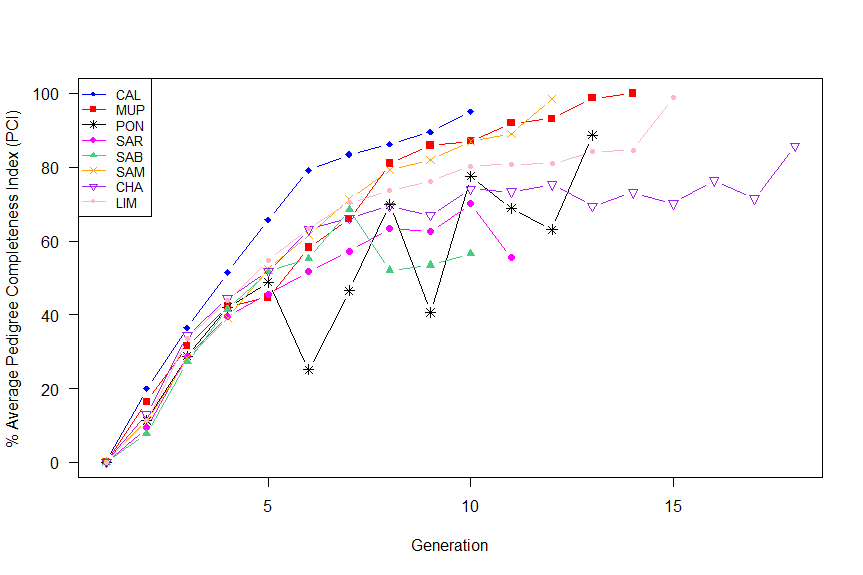

Supplement: Supplementary file 1 [file animals-09-00880-s001.zip › Supp_Fig2.png]

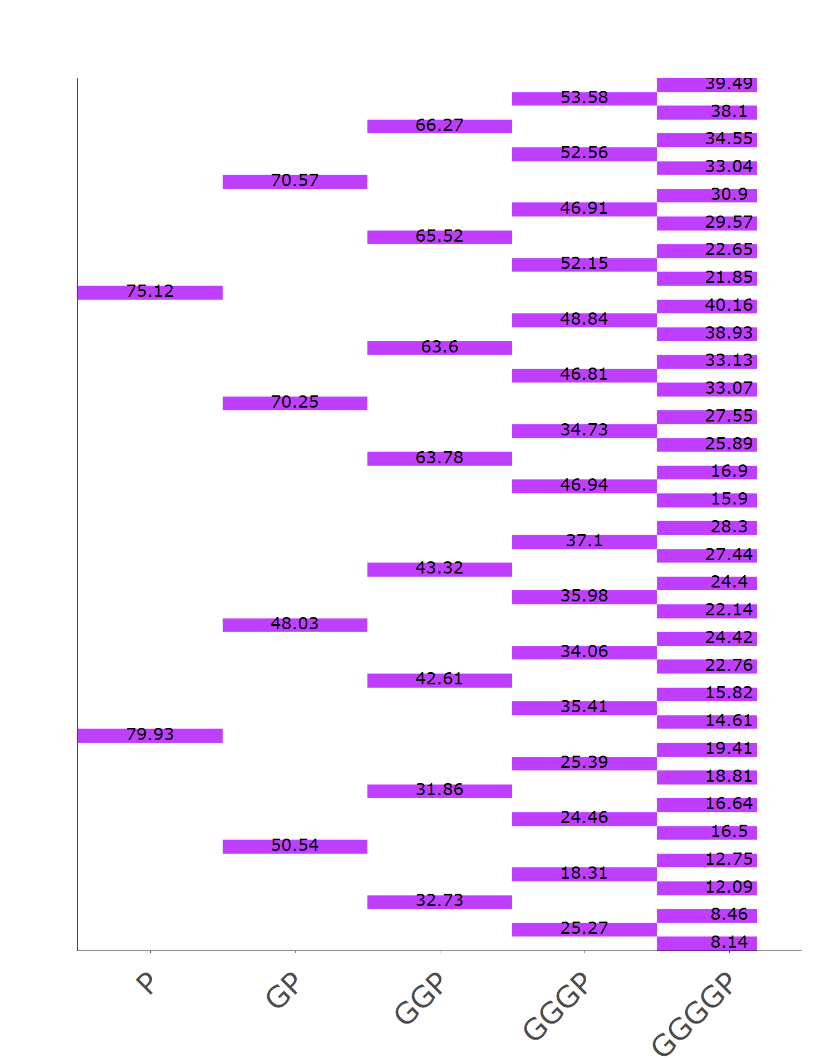

Supplement: Supplementary file 1 [file animals-09-00880-s001.zip › Suppl_F5_CHA.png]
